# Seasonal Phenology and Temporal Niche Differentiation of Dung Beetle Communities in Yak Dung from Alpine Meadows of the Eastern Qilian Mountains

**DOI:** 10.3390/biology15171524

**Published:** 2026-09-03

**Authors:** Liang Mao, Yuping Gou, Shaochong Wei, Haijie Zhao, Jintao Shi, Xiaojun Yu

**Affiliations:** 1 College of Pratacultural Science, Gansu Agricultural University, Lanzhou 730070, China; maolchina@163.com (L.M.); weisc@st.gsau.edu.cn (S.W.); 18393947730@163.com (H.Z.); 15117257996@163.com (J.S.); 2 Forestry and Grassland Bureau of Lintao County, Dingxi 730075, China; 3 College of Plant Protection, Gansu Agricultural University, Lanzhou 730070, China; gouyp@gsau.edu.cn

**Keywords:** Scarabaeoidea, coprophagy, plateau pasture, resource partitioning, β-diversity

## Abstract

Warm-season alpine meadow pastures on the Qinghai–Tibet Plateau receive large inputs of yak dung, yet the temporal organization of dung beetle communities inhabiting this resource remains poorly understood. From May to October 2025, we investigated dung beetle communities inhabiting yak dung in warm-season alpine meadow pastures of the eastern Qilian Mountains. A total of 28,208 beetles were collected, of which 27,068 individuals representing 12 scarabaeoid taxa were retained for community analyses. The community was dominated by *Aphodius*, with *A. sublimbatus* and *A. erraticus* as the dominant species. Community composition, density, biomass, and diversity showed pronounced monthly variation. Both density and biomass peaked in August, reaching 31.44 adults/pat and 407.38 mg/pat, respectively. Hill–Shannon diversity remained relatively high from July to September, whereas Sørensen beta diversity declined from 0.305 in May to 0.090 in August, indicating greater similarity in community composition among transects in August. Species activity timing and niche breadth also varied markedly among months, while mean Pianka niche overlap increased from 0.567 in May to 0.752 in August and subsequently declined. These results indicate seasonal niche differentiation among dung beetle species in both activity timing and resource use, which may reduce prolonged resource-use overlap and thereby facilitate species coexistence. This study provides a basis for understanding the seasonal dynamics, resource use, and coexistence of dung beetle communities in alpine grazing ecosystems.

## Simple Summary

Yak dung can accumulate on grasslands and influence nutrient cycling and pasture health. Dung beetles play an important role in breaking down animal dung, yet little is known about how their communities change throughout the warm season in high-altitude grasslands. In this study, we investigated dung beetle communities living in yak dung from May to October in the eastern Qilian Mountains of the Qinghai–Tibet Plateau. We found significant monthly variation in dung beetle community structure, with the community dominated by *Aphodius* species, particularly *A. sublimbatus* and *A. erraticus*. Beetle density and biomass were highest in August, while diversity remained relatively high from July to September. Different species also differed in when and how broadly they used yak dung resources. These seasonal differences may reduce prolonged overlaps in resource use and help multiple species coexist. Our study improves understanding of how dung beetles contribute to the natural processing of yak dung in alpine grasslands and provides useful information for maintaining healthy grazing ecosystems and improving sustainable pasture management.

## 1. Introduction

The Qilian Mountains represent a critically important ecological region on the northeastern margin of the Qinghai–Tibet Plateau, supporting biodiversity conservation, regional climate regulation, and water retention [1,2]. Alpine meadows, as the dominant ecosystem in the region, are essential for maintaining fragile alpine ecosystem stability and sustaining plateau livestock production [3,4,5]. However, overgrazing and climate change increasingly threaten these grasslands, causing soil erosion, biodiversity loss, and weakened ecosystem functioning [6,7]. Therefore, conserving and sustainably using alpine meadows is central to ecological security in the Qilian Mountains. The area is divided into cold-season and warm-season pastures according to climate, topography, and altitude, and rotational grazing practices are applied to protect grassland resources [8,9]. Dynamic stocking, involving herd expansion during the warm season and livestock off-take during the cold season, is used to maintain forage–livestock balance [10,11]. Nevertheless, with the rapid development of China’s grassland livestock sector, livestock numbers in the alpine meadows of the Qilian Mountains have risen markedly, from roughly 0.7 million head in the 1950s to 1.8 million head in the early 21st century [12].

Research on grazed alpine meadow ecosystems has mainly addressed vegetation phenology [13], soil respiration [14] and microbial community reassembly [15]. The long-term retention of yak dung and the resulting “ecological black hole” effect on grasslands, however, are frequently underestimated [16]. Dung deposition not only physically blankets vegetation but also triggers chlorosis in plants beneath the pat and causes substantial damage to many dicotyledonous species. Moreover, cattle avoid herbage adjacent to dung pats, leading to wasted fodder and reduced grassland production [17,18]. A grazing cow can produce enough dung over 180 days to cover up to 160 m^2^ of pasture [19]. The area of rejected herbage around a pat is approximately 17.5 times that of the pat itself [20]. Animal excreta are estimated to cover about 5% of the annual grazed area of grasslands worldwide [21]. The Qinghai–Tibet Plateau holds roughly 16 million yaks, accounting for 95% of the global yak population [22].

Grazing pressure on available grasslands is estimated at 1.2 yaks/km^2^ [23]. Yak dung pat density can reach up to 5900 pats/ha, with an annual accumulation of 40 million tons of dung [24], covering over 20% of the total grazing area [25]. The large accumulation and slow decomposition of yak dung therefore represent a substantial but often overlooked challenge for alpine meadow ecosystems. In natural grazing systems, dung beetles are key agents of yak dung decomposition: their foraging and nesting activities accelerate dung fragmentation, influence microbial diversity within the pats, promote nutrient mineralization, and ultimately, improve soil fertility [26,27,28]. To mitigate widespread cattle dung accumulation in Australian pastures, the Dung Beetle Program introduced dung beetle species from multiple regions to enhance dung degradation [29]. In the Nebraska Sandhills, USA, dung beetles accounted for 7% of moisture loss and 4% of dry matter loss from cattle dung [30]. In alpine meadows of the Qinghai–Tibet Plateau, yak dung decomposition is positively correlated with the abundance of coprophagous insects within pats [31,32]. Coprophagous scarab beetles also show clear activity rhythms and seasonal patterns; most species in tropical Brazil forage during daytime and crepuscular periods [33], and *A. rectus* has been reported as the dominant taxon in cattle dung in China’s Songnen grassland in April, May, June and September [34].

Despite the extensive warm-season pastures, large yak populations, and substantial dung accumulation in the Qilian Mountains, the monthly dynamics of dung beetle community in yak dung remain poorly understood. We hypothesized that dung beetle community composition and density would show distinct monthly turnover, with diversity and activity peaking during the warmest months, thereby reflecting shifts in dung-resource use by dung beetles. To test this hypothesis, we surveyed adult dung beetles in yak dung during the first ten days of each month from May to October 2025 in warm-season alpine meadow pastures of the Qilian Mountains. Specifically, we aimed to quantify monthly variation in species composition and population dynamics, identify key coprophagous taxa associated with yak dung use, and determine the main temporal window of dung beetle activity. These results provide a foundation for understanding the potential ecological roles of dung beetles in alpine grazing systems.

## 2. Materials and Methods

### 2.1. Study Area

This study was conducted in alpine meadows of the eastern Qilian Mountains, Qinghai–Tibet Plateau, China (37°00′ N–38°20′ N, 101°00′ E–103°00′ E), at a mean altitude exceeding 3000 m a.s.l. The region has a mean annual temperature of 0–0.6 °C and mean annual precipitation of 300–450 mm, with wet summers and autumns and cold, dry winters and springs, which are characteristic of a semi-arid continental plateau climate [35]. Meteorological data on temperature and precipitation during the study period (May–October 2025) were obtained from the China Meteorological Data Service Center. The highest temperatures were recorded in July, while precipitation was predominantly concentrated in June, July, and August (Figure S1). The predominant soil type is subalpine meadow soil. The vegetation is typical of alpine meadow, with dominant species including *Carex alatauensis*, *Elymus nutans*, and *Poa pratensis*. Common associated species are *Bistorta vivipara*, *Thalictrum dioicum*, *Potentilla chinensis*, and *Saussurea japonica*. The growing season typically commences in April, with plants senescing by the end of September [36].

### 2.2. Plot Establishment

**Figure 1:**
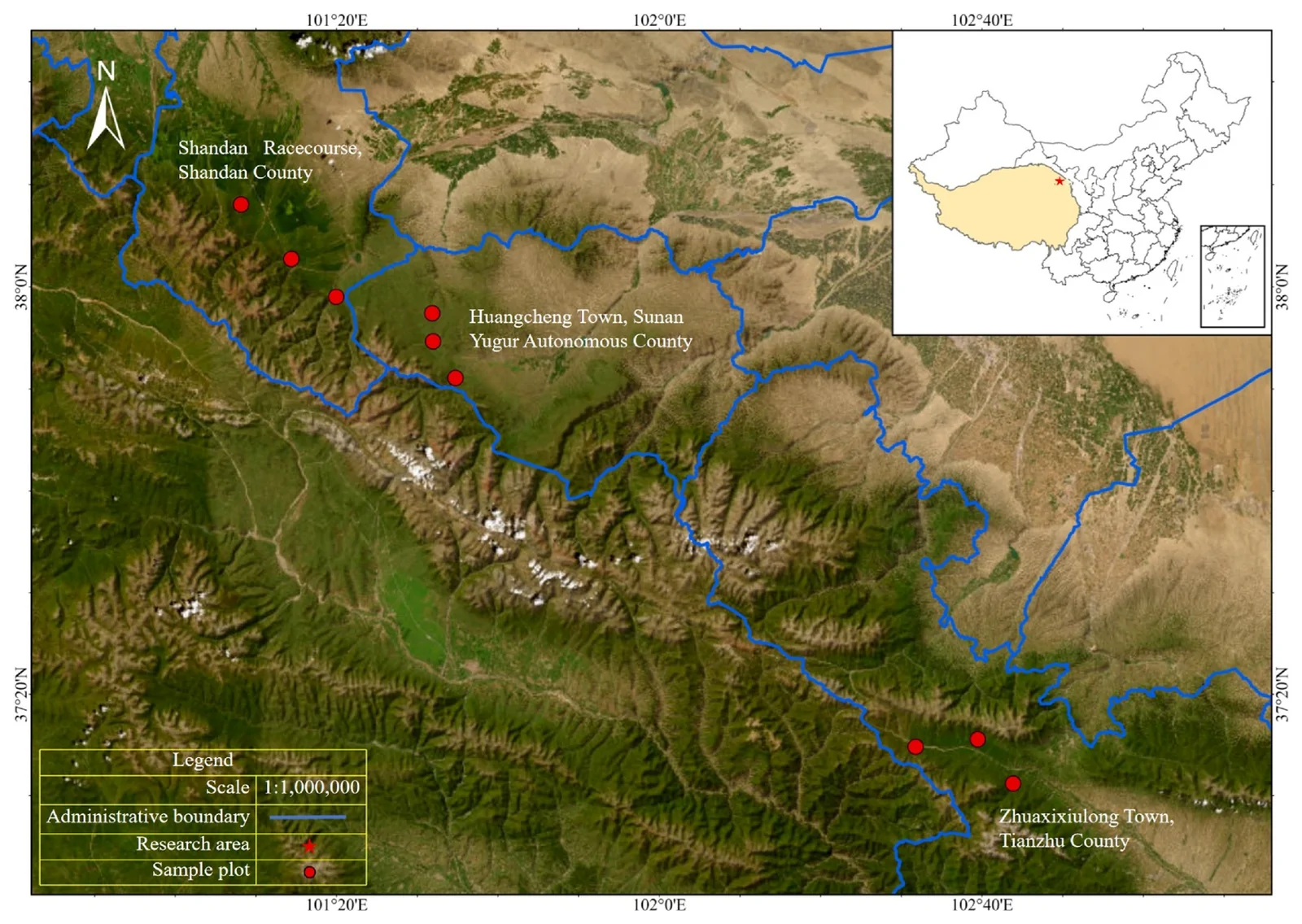
Study area and sampling design. Blue lines indicate administrative boundaries. White letters denote the names of the three sampling areas, and red dots represent the three sampling plots established within each area. The base map of China is sourced from the National Geospatial Information Services Platform (Map Approval No. GS (2023)2767). The red five-pointed star indicates the location of the study area.

This study was conducted at yak-grazed alpine meadow sites that had been used for 5–8 years, with flat terrain (slope < 5°), low shrub cover (<10%), and an elevation of approximately 3100 m a.s.l. Three sampling sites were established at Shandan Horse Farm in Shandan County, Huangcheng Town in Sunan Yugur Autonomous County, and Zhuaxixiulong Township in Tianzhu County. At each site, three sampling plots were established (Figure 1); the coordinates and elevations of all nine sampling plots are provided in Table S1.

### 2.3. Sampling Methods

Beetles were sampled during the first ten days of each month from May to October 2025. In each of the nine plots, three parallel 100 m transects were established at 50 m intervals, and ten yak dung pats were selected along each transect. The selected pats were flattened, pat-shaped dung, 15–25 cm in diameter and 5–10 cm thick, with an air-dried surface and a soft interior. Each dung pat, together with the underlying surface soil, was collected in a plastic bag following Jiang and Zhou [18]. Outdoors, each sample was placed in a 40-mesh nylon bag, manually broken apart, and washed in water for approximately 1 min. The residue was transferred to a bucket of water, mixed, and allowed to settle for 3–5 min. Floating debris was collected with a 40-mesh sieve, and dung beetles were separated manually with forceps. When hand sorting was insufficient, the remaining material was processed using a grassland soil insect collector based on the Tullgren extraction method [37,38]. Beetles from each dung pat were preserved separately in 75% ethanol for subsequent identification and counting in the laboratory [39]. In total, 1620 yak dung pats were examined. Based on 30 randomly selected pats, mean fresh weight, dry weight, and water content were 838.47 g, 264.10 g, and 68%, respectively.

### 2.4. Insect Identification

Beetles recovered from dung pats were examined and photographed using a Motic SMZ-171 stereomicroscope and Moticam Pro 285A camera. Specimens were identified using standard taxonomic references, including *Chinese Insects Illustrated* [40], *Colour Illustration of Scarab in Northern China* [41], *Fauna of the Beetles from Ningxia*, *China* [42], *The Scarabaeoidea Chronicles of Mount Wuyi* [43], and *Ecology and Evolution of Dung Beetles* [44,45]. Whenever possible, specimens were identified to species level; otherwise, they were assigned to genus and treated as distinct morphospecies. Representative voucher specimens of all identified species and morphospecies were deposited in the Laboratory of Grassland Science, College of Grassland Science, Gansu Agricultural University.

### 2.5. Metric Calculation

For analyses of dung beetle community composition, density, biomass, diversity, and niche characteristics, “dung beetles” were restricted to taxa within the superfamily Scarabaeoidea, whereas non-scarabaeoid taxa were excluded from these analyses (Table S2).

For each taxon and month in which it occurred, individual dry biomass was estimated as the mean dry mass of 30 randomly selected adults (mg/adult). Specimens were dried at 45 °C for 48 h, equilibrated to room temperature in a desiccator, and weighed with a Sartorius BCE224-1CCN analytical balance (precision: 0.1 mg) [46]. Community density was expressed as the total number of adult dung beetles per yak dung pat (adults/pat) [47]. Community dry biomass was calculated as the sum, across taxa, of the product of mean individual dry biomass and abundance within each yak dung pat (mg/pat).

Alpha diversity was characterized by Hill–Shannon diversity ($D_{1}$) and observed species richness (S) [48,49]. Sørensen dissimilarity ($\beta_{sor}$) was partitioned into turnover ($\beta_{sim}$) and nestedness component ($\beta_{sne}$) [50,51,52]. Levins niche breadth ($B_{i}$) and Pianka niche overlap ($O_{ik}$) were used to characterize niche structure [53,54].

### 2.6. Statistical Analysis

Maps were produced using ArcGIS 10.8 (ESRI, Redlands, CA, USA), and figures were generated using OriginPro 2024 (OriginLab Corp., Northampton, MA, USA). All statistical analyses were performed in R version 4.4.2 (R Core Team, Vienna, Austria). Seasonal abundance patterns of individual taxa were summarized by average-linkage hierarchical clustering based on Bray–Curtis dissimilarity of monthly relative-abundance profiles using vegan 2.7.3. Community compositional variation among sample units was visualized by non-metric multidimensional scaling (NMDS) using Bray–Curtis dissimilarity and tested by PERMANOVA with 9999 permutations restricted within repeatedly sampled transects; homogeneity of multivariate dispersion was assessed using PERMDISP. Convex hulls in the NMDS ordination were used only for visualization.

Monthly differences in individual dry biomass within taxa were tested using Gaussian linear models followed by Tukey-adjusted pairwise comparisons. Community counts were analyzed with a negative-binomial generalized linear mixed-effects model in lme4 2.0.1 after Poisson overdispersion was detected, with month as a fixed effect, transect as a random intercept, and the log number of sampled dung pats as an offset; density is reported as adults/pat. Community dry biomass was analyzed with a Gaussian linear mixed-effects model using month as a fixed effect and transect as a random intercept. Observed species richness and Hill–Shannon diversity were analyzed with Gaussian linear models including transect as a fixed blocking factor. Pairwise comparisons used Tukey-adjusted estimated marginal means in emmeans 2.0.3; Satterthwaite degrees of freedom for the mixed model were obtained with LmerTest 3.2.1. Effect sizes were reported as marginal *R*^2^ for the density model and partial *η*^2^ for Gaussian models, and model adequacy was assessed using residual diagnostics.

Within-month beta diversity was quantified using Sørensen dissimilarity and partitioned into turnover and nestedness components from pairwise comparisons among transects. Levins niche breadth and Pianka niche overlap were calculated separately for each month from species abundance distributions across transects; niche metrics were undefined when a species was absent.

## 3. Results

### 3.1. Morphological Characteristics and Adult Dry Biomass

**Figure 2:**
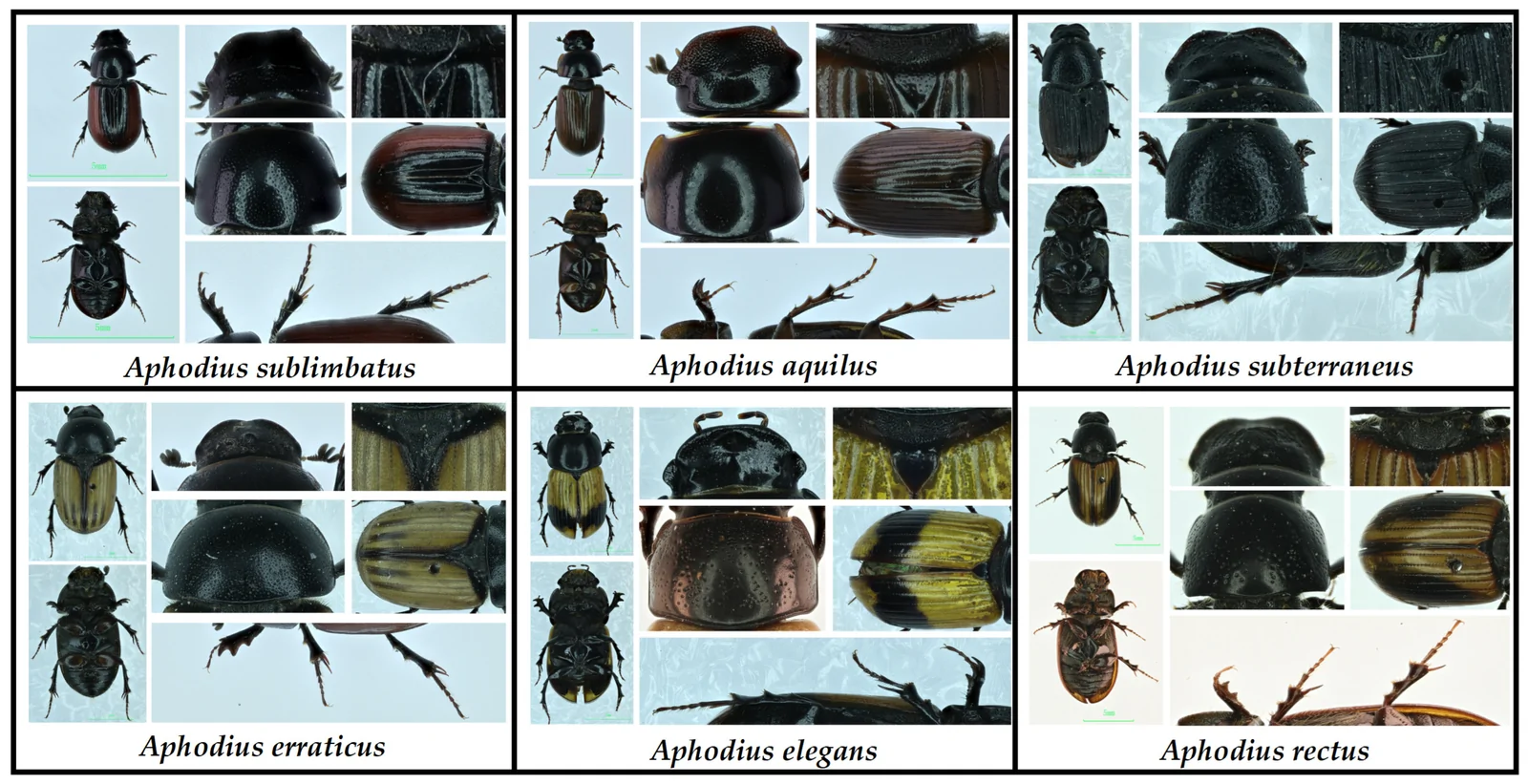
Representative adult scarabaeoid dung beetles inhabiting yak dung.

A total of 28,208 beetles representing 17 species across 8 genera and 5 families were collected from yak dung pats. Of these, 27,068 individuals belonging to 12 scarabaeoid taxa were retained for community analyses. Representative images of the six dominant taxa are presented in Figure 2, while a complete plate of diagnostic images for all 17 species is provided in Supplementary Figure S2.

Individual adult dry biomass varied significantly among months in 11 of the 12 scarabaeoid taxa (Table 1), whereas no monthly difference was detected for *Onthophagus gibbulus*. For most taxa, the highest mean dry biomass occurred in August or September.

### 3.2. Temporal Dynamics of Community Structure

**Figure 3:**
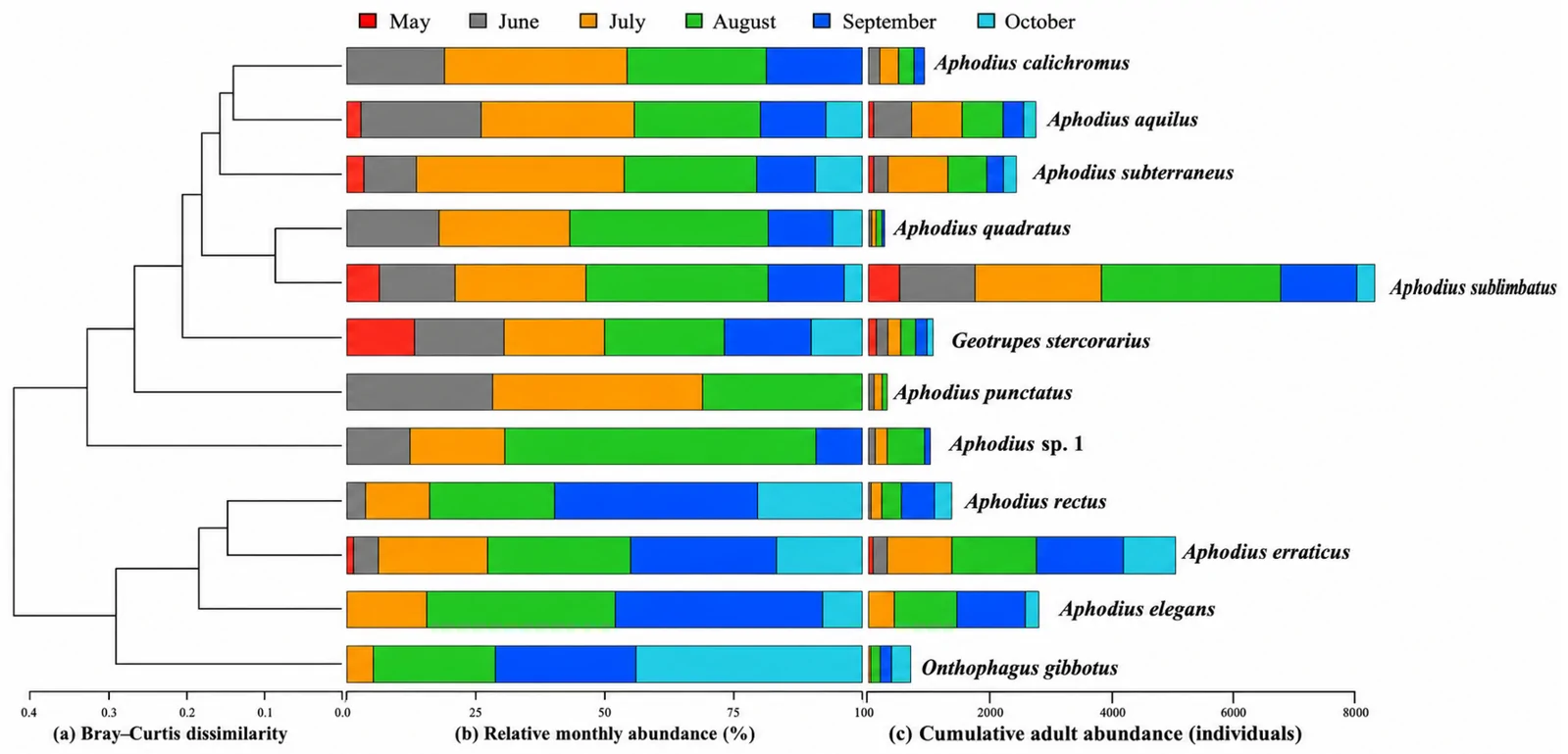
Seasonal abundance patterns and hierarchical clustering of 12 scarabaeoid dung beetle taxa in yak dung. (**a**) Average-linkage clustering based on Bray–Curtis dissimilarity of monthly relative abundance profiles. (**b**) Relative monthly abundance of each taxon, with each bar summing to 100%. (**c**) Cumulative adult abundance partitioned by month.

Hierarchical clustering based on Bray–Curtis dissimilarity of monthly relative abundance profiles revealed clear differences in seasonal occurrence among the 12 scarabaeoid taxa (Figure 3a). Several species, including *Aphodius punctatus*, *A. aquilus*, *A. subterraneus*, and *A. calichromus*, were concentrated mainly from June to August, whereas *A. erraticus*, *A. rectus*, *A. elegans*, and *Onthophagus gibbulus* showed relatively later seasonal activity, with higher proportions in August-October. *Aphodius* sp. 1 exhibited a distinct August-centered pattern.

Cumulative abundance was strongly dominated by a few taxa (Figure 3c). *A. sublimbatus* was the most abundant species (8309 individuals), followed by *A. erraticus* (5063), *A. elegans* (2815), *A. aquilus* (2754), and *A. subterraneus* (2439); together, these five taxa accounted for 79% of the 27,068 scarabaeoid beetles retained for community analyses.

**Figure 4:**
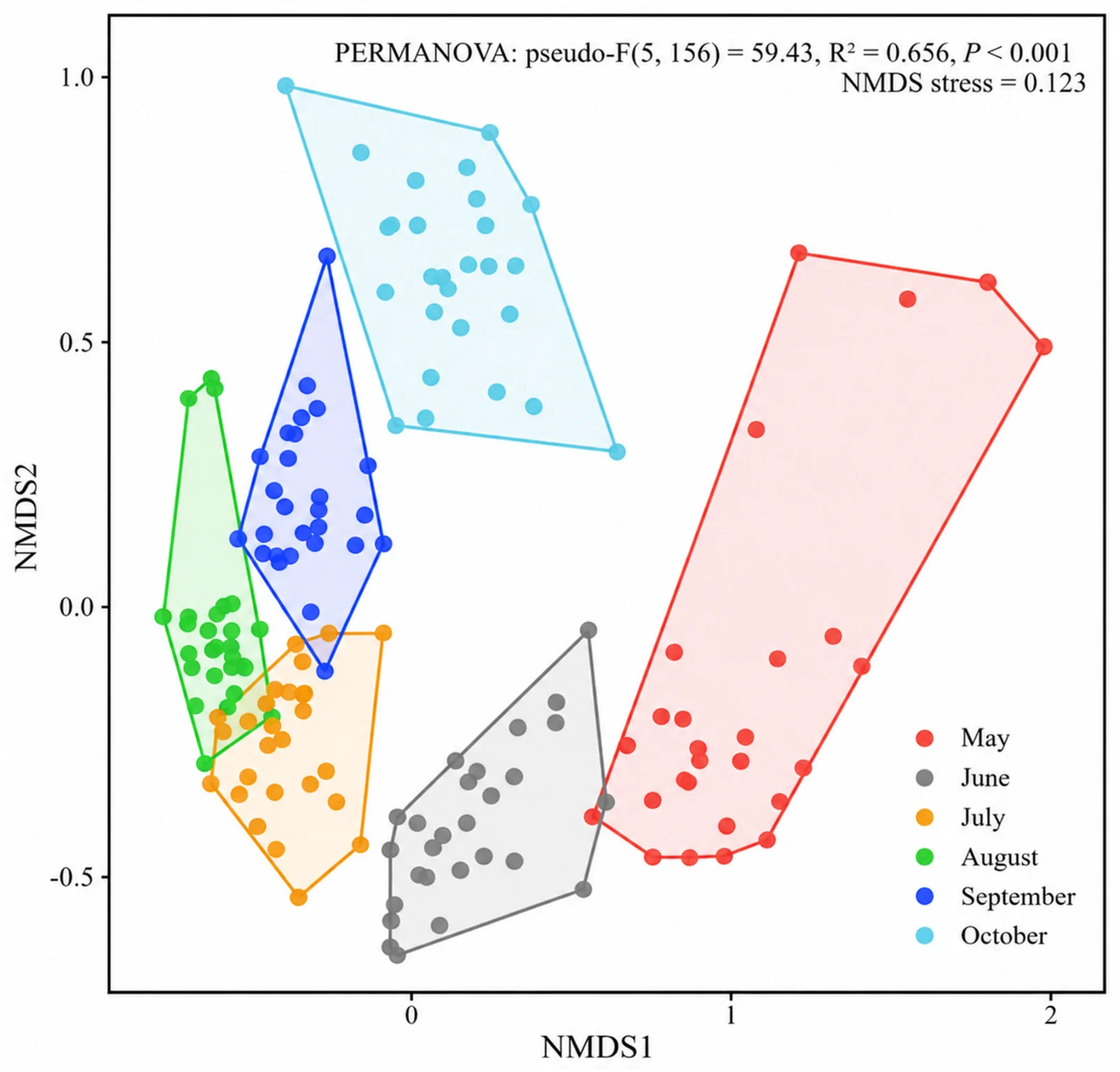
NMDS ordination of dung beetle communities from May to October based on Bray–Curtis dissimilarity among 162 transect-month samples comprising 12 scarabaeoid taxa.

NMDS ordination based on Bray–Curtis dissimilarity revealed pronounced temporal structuring of the dung beetle community across the six sampling months (Figure 4). The two-dimensional solution showed an acceptable fit (stress = 0.123). PERMANOVA, with permutations restricted within repeatedly sampled transects, indicated a significant effect of month on community composition (pseudo-F_5,156_ = 59.43, *R*^2^ = 0.656, *p* < 0.001). However, multivariate dispersion also differed among months (PERMDISP: F_5,156_ = 3.58, *p =* 0.004), indicating that the PERMANOVA result may partly reflect differences in within-month dispersion as well as shifts in community composition. The ordination nevertheless showed a clear temporal progression in community structure from May to October.

**Figure 5:**
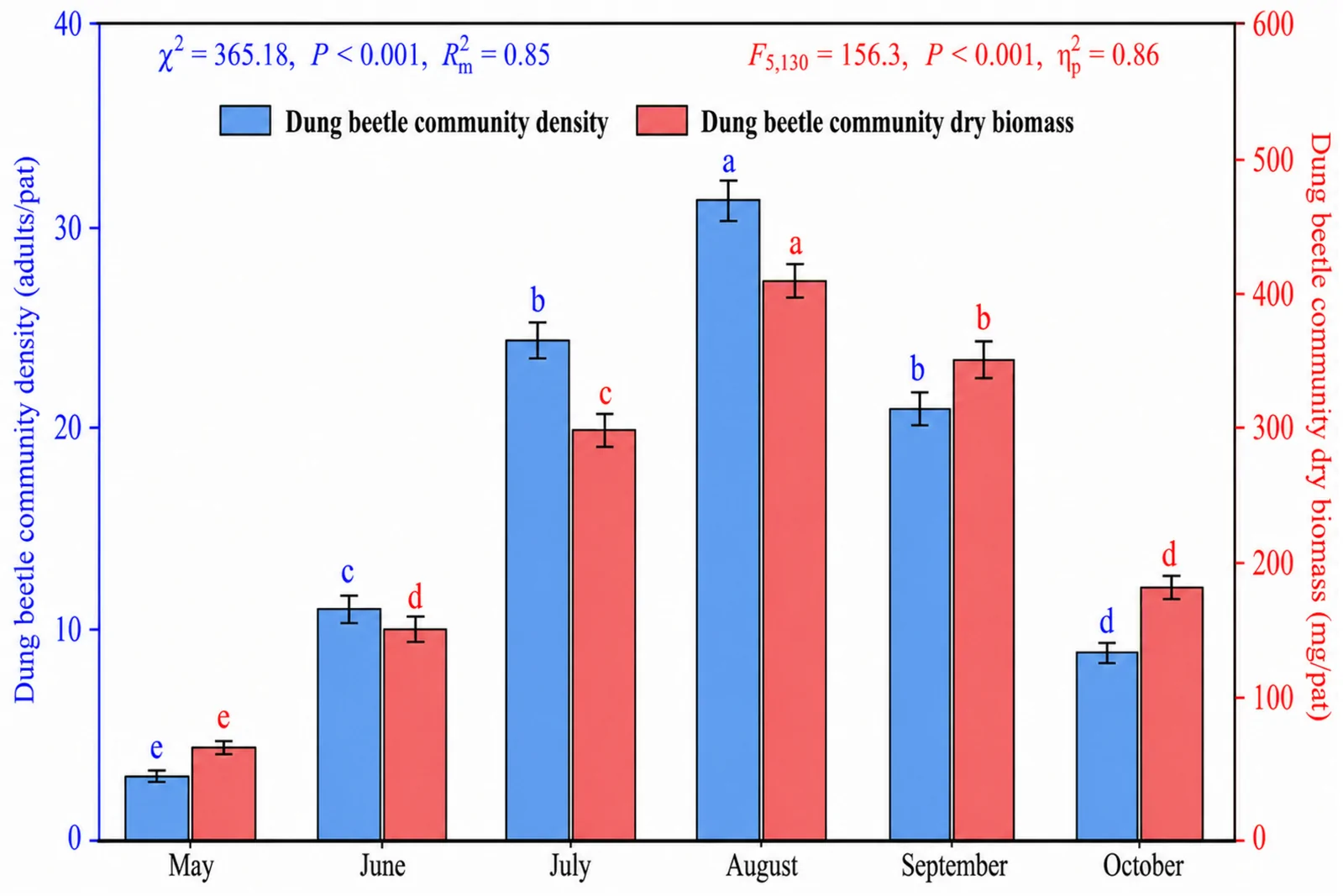
Monthly variation in dung beetle community density and dry biomass in yak dung. Bars show mean ± SE (*n* = 27 transects per month). Density and biomass were analyzed using negative-binomial and Gaussian mixed-effects models, respectively. Different lowercase letters indicate significant differences among months (*p* < 0.05).

Dung beetle community density and dry biomass varied significantly among months (Figure 5). Community density increased from 3.24 ± 0.25 adults/pat in May to a maximum of 31.44 ± 1.08 adults/pat in August and subsequently declined to 9.21 ± 0.39 adults/pat in October. The negative-binomial generalized linear mixed-effects model revealed a significant effect of month on community density (*x*^2^_5_ = 365.18, *p* < 0.001, marginal *R*^2^ = 0.855). Tukey-adjusted pairwise comparisons showed that density was highest in August and lowest in May, whereas July and September did not differ significantly (*p* > 0.05). Community dry biomass followed a similar seasonal pattern, increasing from 68.62 ± 4.31 mg/pat in May to 407.38 ± 11.42 mg/pat in August before declining thereafter. Month had a significant effect on community dry biomass (F_5,130_ = 156.30, *p* < 0.001, partial *η*^2^ = 0.857). Biomass was highest in August and lowest in May, whereas June and October did not differ significantly (*p* > 0.05).

### 3.3. Temporal Dynamics of Diversity

**Figure 6:**
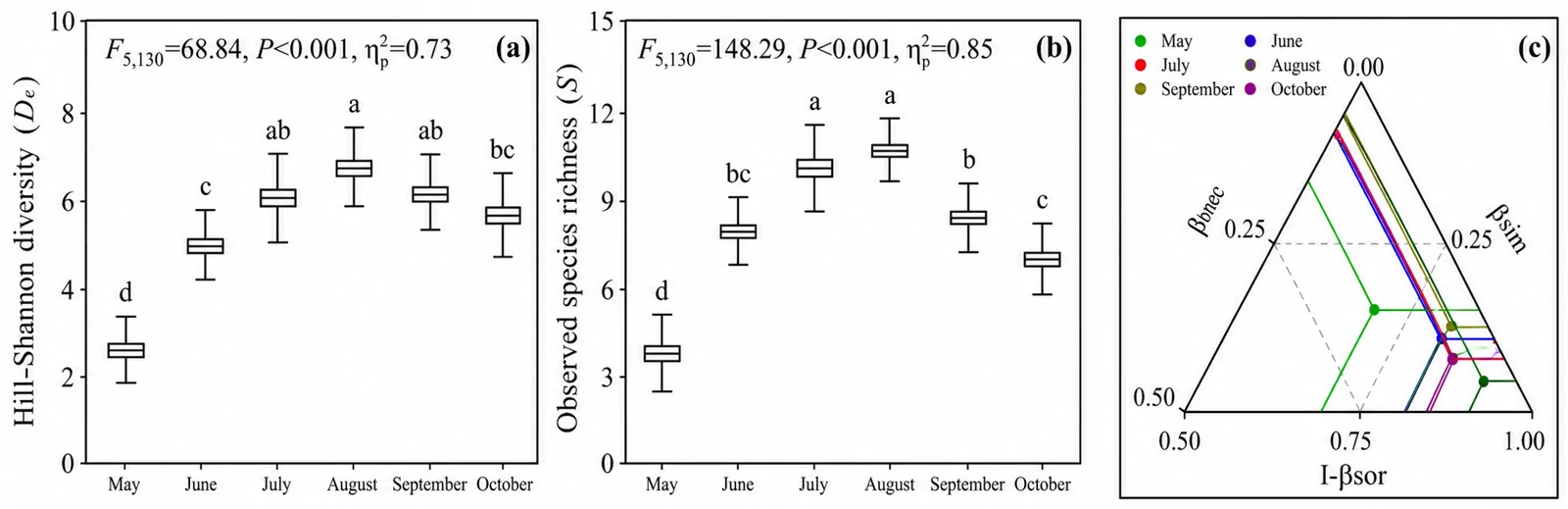
Monthly variation in dung beetle alpha and beta diversity. (**a**) Hill–Shannon diversity (*D*_1_); (**b**) observed species richness (*S*); (**c**) Sørensen beta diversity partitioned into turnover (*βsim*) and nestedness (*βsne*) components, with 1 − *βsor* representing community similarity. Boxplots show medians, interquartile ranges, and ranges; different lowercase letters denote significant monthly differences (Tukey-adjusted, *p* < 0.05). Beta-diversity values are means of pairwise comparisons among 27 transects.

Dung beetle alpha diversity varied significantly among months (Figure 6a,b). Hill–Shannon diversity increased from 2.59 ± 0.14 in May to 6.77 ± 0.16 in August and remained relatively high through September before declining in October. Month had a significant effect on Hill–Shannon diversity (F_5,130_ = 68.84, *p* < 0.001, partial *η*^2^ = 0.73). Tukey-adjusted comparisons showed that July, August, and September did not differ significantly from one another, whereas May had significantly lower diversity than all other months.

Observed species richness showed a similar seasonal pattern, increasing from 3.48 ± 0.20 species per sample in May to 10.78 ± 0.19 in August (F_5,130_ = 148.29, *p* < 0.001, partial *η*^2^ = 0.85). Species richness was highest in July and August, with no significant difference between these two months, and declined thereafter.

Within-month Sørensen beta diversity was highest in May (*βsor* = 0.305) and lowest in August (*βsor* = 0.090), corresponding to community similarities of 0.695 and 0.910, respectively (Figure 6c). Turnover exceeded nestedness-resultant dissimilarity in June (*βsim* = 0.105 vs. *βsne* = 0.080) and was especially pronounced in September (0.124 vs. 0.057), whereas the two components were similar in May, July, August, and October.

### 3.4. Temporal Dynamics of Niche Structure

**Figure 7:**
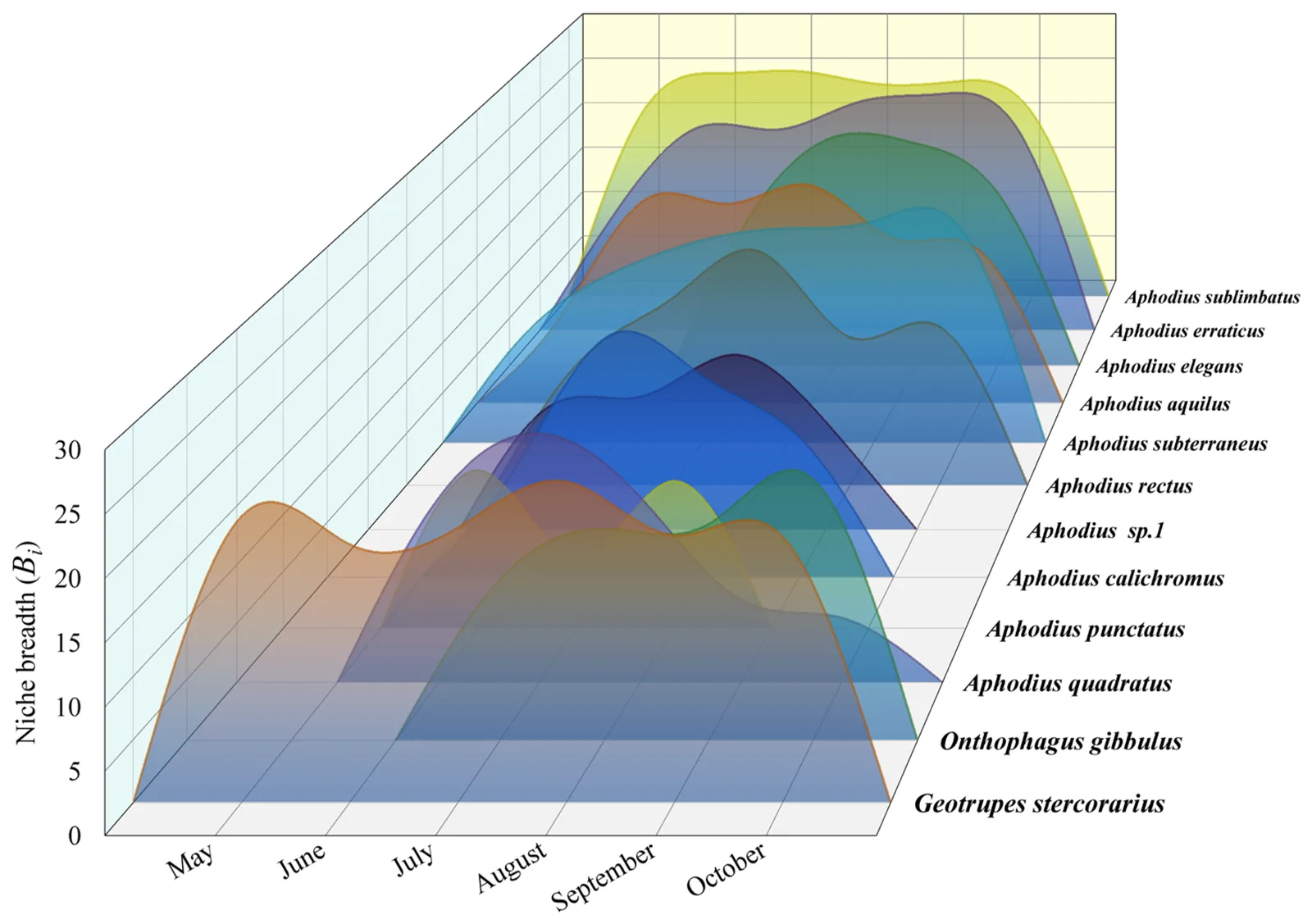
Monthly variation in Levins niche breadth (*B_i_*) of dung beetle taxa in yak dung. Values were calculated from proportional abundance across 27 transects, with higher *B_i_* indicating broader spatial niche use.

Levins niche breadth varied markedly among dung beetle taxa and across months (Figure 7). *Aphodius sublimbatus* maintained a broad niche from May to October and peaked in July (*B_i_* = 25.01), whereas *Geotrupes stercorarius* peaked in August (*B_i_* = 25.42). The niche breadth of *A. erraticus* increased from 13.34 in May to 25.52 in September, and *A. elegans* and *Onthophagus gibbulus* showed later-season expansion. In contrast, *A. punctatus* and *Aphodius* sp. 1 occurred over shorter seasonal windows, whereas *A. quadratus* showed a marked decline in niche breadth after July. These patterns indicate pronounced seasonal shifts in the spatial use of yak dung resources.

**Figure 8:**
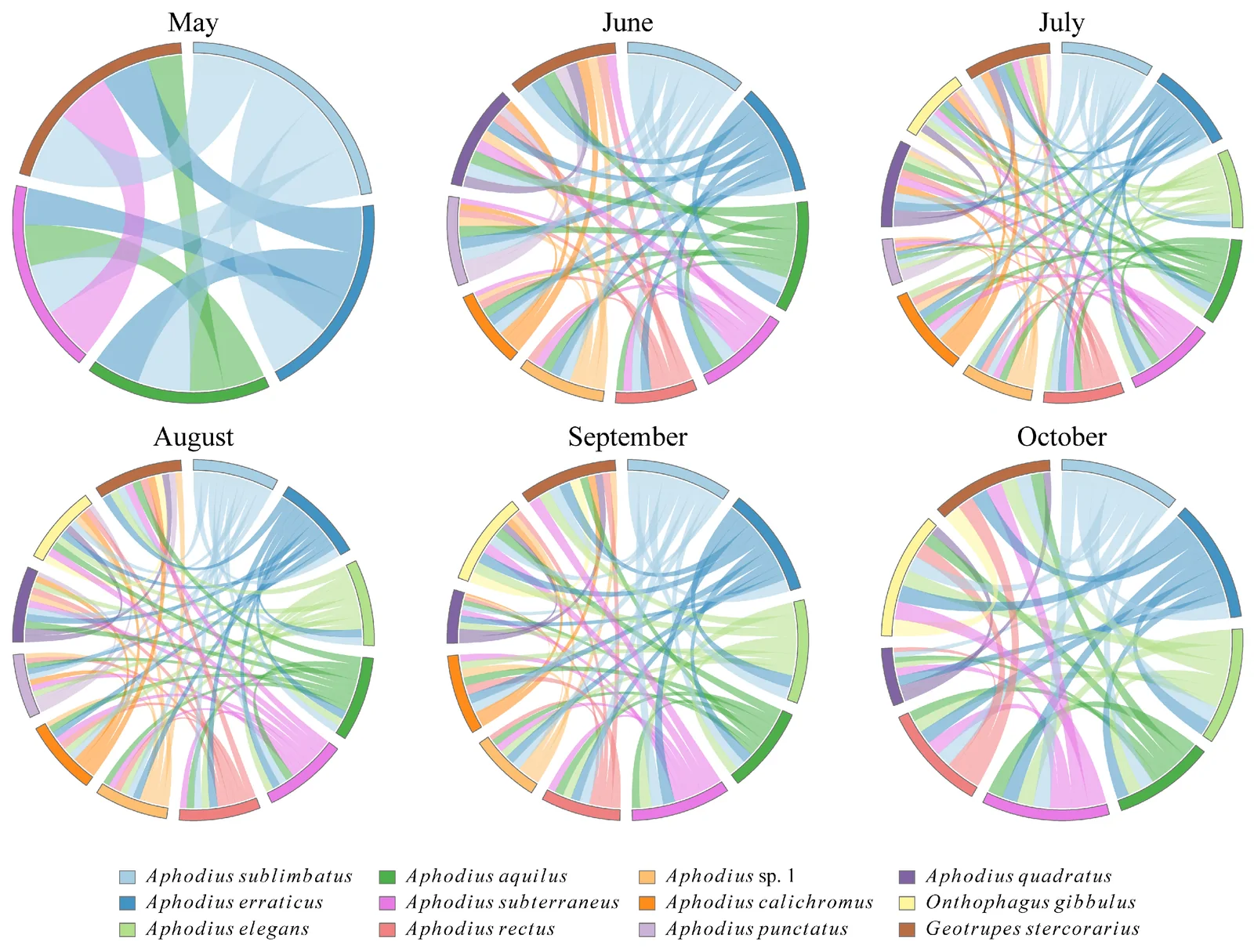
Monthly Pianka niche overlap (*O_ik_*) among scarabaeoid dung beetles in yak dung. Overlap was calculated from proportional abundance across 27 transects; ribbon width indicates the magnitude of spatial niche similarity.

Pianka niche overlap varied markedly among species pairs and months (Figure 8). Mean pairwise overlap increased from 0.567 in May to 0.752 in August, when 65 of the 66 defined species pairs had overlap values ≥ 0.5; it then declined to 0.627 in September and was 0.635 in October. The species pair with the highest overlap differed among months: *Aphodius sublimbatus* and *Geotrupes stercorarius* in May (*O_ik_* = 0.779), *A. sublimbatus* and *A. aquilus* in June (*O_ik_* = 0.895), *A. sublimbatus* and *A. calichromus* in July (*O_ik_* = 0.898), and *A. erraticus* and *G. stercorarius* in August (*O_ik_* = 0.922). Across the entire sampling period, the highest overlap occurred between *A. erraticus* and *A. elegans* in September (*O_ik_* = 0.926), whereas the lowest occurred between *A. calichromus* and *A. quadratus* (*O_ik_* = 0.104). Overall, niche overlap among species was generally high in August, whereas variation in overlap among species pairs increased thereafter.

## 4. Discussion

### 4.1. Seasonal Environmental Variation and Dung Beetle Community Restructuring

Our results showed pronounced seasonal variation in dung beetle communities associated with yak dung. *Aphodius sublimbatus* and *A. erraticus* were the dominant species and maintained relatively high activity over extended periods. In contrast, *A. elegans* and *Onthophagus gibbulus* were more active later in the warm season, whereas *A. punctatus* and *Aphodius* sp.1 occurred over relatively short periods. Community density and biomass increased markedly during the warm season. Although multivariate dispersion differed among months, the NMDS ordination and changes in species abundance still revealed a clear seasonal pattern in community composition.

These changes may be associated with the strong seasonality of the alpine meadow environment. Low temperatures can constrain insect activity and alter seasonal activity through their effects on development and post-overwintering energy balance [54]. Laini et al. [55] found that temperature and habitat type were important factors shaping alpine dung beetle community composition. As temperature increased and moisture conditions improved during the warm season, conditions became more favorable for dung beetle activity, feeding, and reproduction. The continuous availability of fresh yak dung during grazing also provided food and breeding resources [56].

Similar seasonal patterns have been reported in other mountain and temperate grassland ecosystems. Mamantov and Sheldon [57] documented pronounced seasonality in dung beetle abundance and distribution in Great Smoky Mountains National Park, USA, while studies from Xinjiang grasslands also reported increased dung beetle activity during the warm season [58]. In the present study, seasonal changes occurred mainly within a community dominated by *Aphodius*, with *A. sublimbatus*, *A. erraticus*, and *A. elegans* differing in both activity period and relative dominance. This suggests that seasonal community dynamics may differ among regions depending partly on species composition.

Seasonal differences among species may also be related to variation in life-history traits. *A. sublimbatus* and *A. erraticus* remained active for relatively long periods, whereas *A. elegans* and *O. gibbulus* became more active in later months, and *A. punctatus* and *Aphodius* sp. 1 had shorter occurrence periods. These patterns indicate that species do not respond to seasonal change in the same way. Lobo and Cuesta [59] likewise showed that dung beetle activity timing can vary seasonally. Thus, the interspecific differences observed here may reflect the combined influence of seasonal environmental variation and species-specific life histories.

### 4.2. Seasonal Variation in Community Diversity and Spatial Heterogeneity

Our results showed that dung beetle community diversity in yak dung from the alpine meadows of the Qilian Mountains followed a “simple–complex–simple” pattern from May to October. This finding is consistent with studies in the Alps by Errouissi et al. [60] and Tocco and Villet [61], in which dung beetle communities transitioned from a simple structure dominated by large tunnelers to a complex structure characterized by an outbreak of small endocoprid species, and then returned to a simple structure dominated by large tunnelers.

Seasonal changes in dung beetle α diversity were not fully synchronized with changes in community density. This may be because community density is strongly influenced by fluctuations in dominant species, whereas Hill–Shannon diversity depends on both species richness and the distribution of relative abundances [48,49]. Similarly, the community in our study underwent clear seasonal restructuring, but the changes occurred mainly among *Aphodius* species. Differences in their activity periods and relative dominance may therefore contribute to contrasting seasonal patterns in community abundance, diversity, and composition.

Partitioning of Sørensen dissimilarity showed that within-month community differences were jointly attributable to species turnover and nestedness-resultant components, with their relative contributions varying among months [50,51]. This indicates that seasonal community change involves not simply gains or losses in species number but also shifts in species occurrence and relative dominance. Thus, as in other mountain ecosystems, seasonal variation in dung beetle diversity in alpine meadows may largely reflect continuous changes in species activity periods and dominance, with seasonal shifts among *Aphodius* species being particularly evident in the present community.

### 4.3. Seasonal Differences in Activity and Niche Differentiation

Activity peaks were not fully synchronized among dung beetle species, indicating clear differences in the timing of yak dung resource use. Some species remained active over relatively long periods, whereas others were concentrated in particular months. Such seasonal differences in activity may reduce prolonged simultaneous use of yak dung by different species and thereby favor coexistence [62,63].

Levins niche breadth varied markedly among months, indicating seasonal changes in species resource-use breadth. Some species maintained relatively broad niches over extended periods, whereas others showed high niche breadth only in particular months, suggesting that species differed in their responses to seasonal conditions. Pianka niche overlap also varied among months. Overall overlap was relatively high during the middle of the warm season, whereas greater variation among species pairs emerged thereafter, indicating that similarity in resource-use patterns was not constant through time. Importantly, high niche overlap indicates similarity in resource use rather than competition intensity itself [64].

In addition to differences in activity timing, functional differences may further contribute to resource-use differentiation among species. The community was dominated by endocoprid *Aphodius* species, whereas *Geotrupes stercorarius* and *Onthophagus gibbulus* represented paracoprid tunnelers; no telecoprid rollers were recorded (Table S2). Endocoprids feed and remain primarily within the dung pat, whereas paracoprids transport dung below ground. These contrasting feeding and dung-processing strategies may therefore generate additional differences in resource use [65]. Together, variation in functional strategy and seasonal activity timing suggests that dung beetle species associated with yak dung differ in both how and when they use dung resources.

## 5. Conclusions

This study showed pronounced seasonal variation in dung beetle communities inhabiting yak dung in alpine meadows of the eastern Qilian Mountains. The community was dominated by *Aphodius,* with *A*. *sublimbatus* and *A*. *erraticus* as the dominant species. Activity periods differed among species, and this seasonal asynchrony may reduce prolonged overlap in yak dung resource use, thereby potentially alleviating resource competition and facilitating species coexistence. These findings provide a basis for understanding the seasonal dynamics and resource-use differentiation of dung beetle communities in alpine grazing systems. Future studies should further compare feeding and dung-processing behaviors among functional groups to evaluate their roles in resource partitioning and species coexistence.

## Figures and Tables

**Table 1:** Temporal variation in adult dry biomass (mg/adult) of scarabaeoid dung beetles inhabiting yak dung.

Species	May	June	July	August	September	October
*Aphodius sublimbatus*	2.1 ± 0.1 d	2.3 ± 0.1 cd	2.6 ± 0.1 bc	2.7 ± 0.1 ab	2.9 ± 0.1 a	2.8 ± 0.1 ab
*Aphodius aquilus*	16.3 ± 0.2 d	16.9 ± 0.3 cd	17.4 ± 0.3 c	18.3 ± 0.3 b	20.2 ± 0.3 a	19.1 ± 0.3 b
*Aphodius subterraneus*	9.8 ± 0.3 b	10.4 ± 0.3 ab	10.8 ± 0.2 ab	11.0 ± 0.3 a	11.4 ± 0.4 a	10.9 ± 0.3 ab
*Aphodius erraticus*	11.5 ± 0.5 c	11.8 ± 0.4 c	12.4 ± 0.3 bc	13.4 ± 0.2 ab	14.3 ± 0.3 a	13.9 ± 0.3 ab
*Aphodius elegans*	-	-	15.9 ± 0.4 c	17.3 ± 0.5 b	19.2 ± 0.4 a	18.1 ± 0.7 ab
*Aphodius rectus*	-	3.1 ± 0.1 b	3.2 ± 0.1 ab	3.4 ± 0.1 a	3.5 ± 0.1 a	3.3 ± 0.1 ab
*Onthophagus gibbulus*	-	-	32.7 ± 0.8 a	33.1 ± 0.9 a	34 ± 0.7 a	33.3 ± 1 a
*Aphodius* sp. 1	-	0.9 ± 0.1 b	1.0 ± 0.1 ab	1.1 ± 0.1 a	1.0 ± 0.1 ab	-
*Aphodius calichromus*	-	5.7 ± 0.2 c	6.0 ± 0.2 bc	6.9 ± 0.2 a	6.4 ± 0.2 ab	-
*Geotrupes stercorarius*	105.5 ± 2.5 c	112.7 ± 2.1 bc	119.3 ± 3.2 b	140.7 ± 5 a	152.2 ± 5.8 a	143.3 ± 4.6 a
*Aphodius punctatus*	-	2.4 ± 0.1 b	2.8 ± 0.1 a	2.6 ± 0.1 a	-	-
*Aphodius quadratus*	-	13.7 ± 0.5 c	14.2 ± 0.3 c	15.7 ± 0.6 b	17.4 ± 0.4 a	16.6 ± 0.4 ab

Values are presented as mean ± SE. Different lowercase letters within a row indicate significant differences in adult dry biomass among sampling months (*p* < 0.05).

## Data Availability

Data will be made available on request.

## Supplementary Materials

The following supporting information can be downloaded at: https://www.mdpi.com/article/10.3390/biology15171524/s1, Table S1: Coordinates and elevations of the nine sampling plots; Table S2: Ecological attributes, life-history information, and analytical inclusion status of the 17 beetle taxa recorded in yak dung [41,42,43,66,67]; Figure S1: Daily mean air temperature and daily precipitation from May to October 2025 at the study site; Figure S2: Diagnostic images of the complete dung-associated beetle voucher inventory.
