# Supplementary material for: Seasonal Phenology and Temporal Niche Differentiation of Dung Beetle Communities in Yak Dung from Alpine Meadows of the Eastern Qilian Mountains
Source: Biology (Basel). 2026 Sep 3;15(17):1524. doi: 10.3390/biology15171524 (PMC13565680; doi:10.3390/biology15171524)
Supplement: Supplementary file 1 [file biology-15-01524-s001.zip › biology-4497016-supplementary.pdf]

Supplementary Materials

Table S1 Coordinates and elevations of the nine sampling plots.

Sampling plots	coordinates	Altitude(m)
Shandan Racecourse, Shandan County	38°8′3″ N 101°8′9″ E	3098
	38°2′43″ N 101°14′23″ E	3189
	37°59′2″ N 101°19′57″ E	3003
Huangcheng Town, Sunan Yugur Autonomous County	37°57′25″ N 101°31′52″ E	3297
	37°54′40″ N 101°31′58″ E	3104
	37°51′5″ N 101°34′44″ E	3020
Zhuaxixiulong Town, Tianzhu County	37°15′7″ N 102°31′55″ E	3282
	37°15′33″ N 102°39′28″ E	3211
	37°11′10″ N 102°43′51″ E	3054

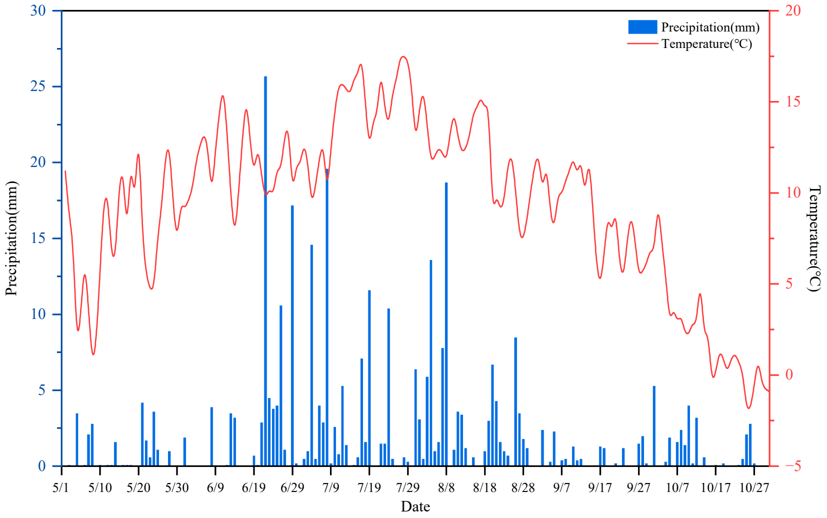

Figure S1 Daily mean air temperature and daily precipitation from May to October 2025 at the study site

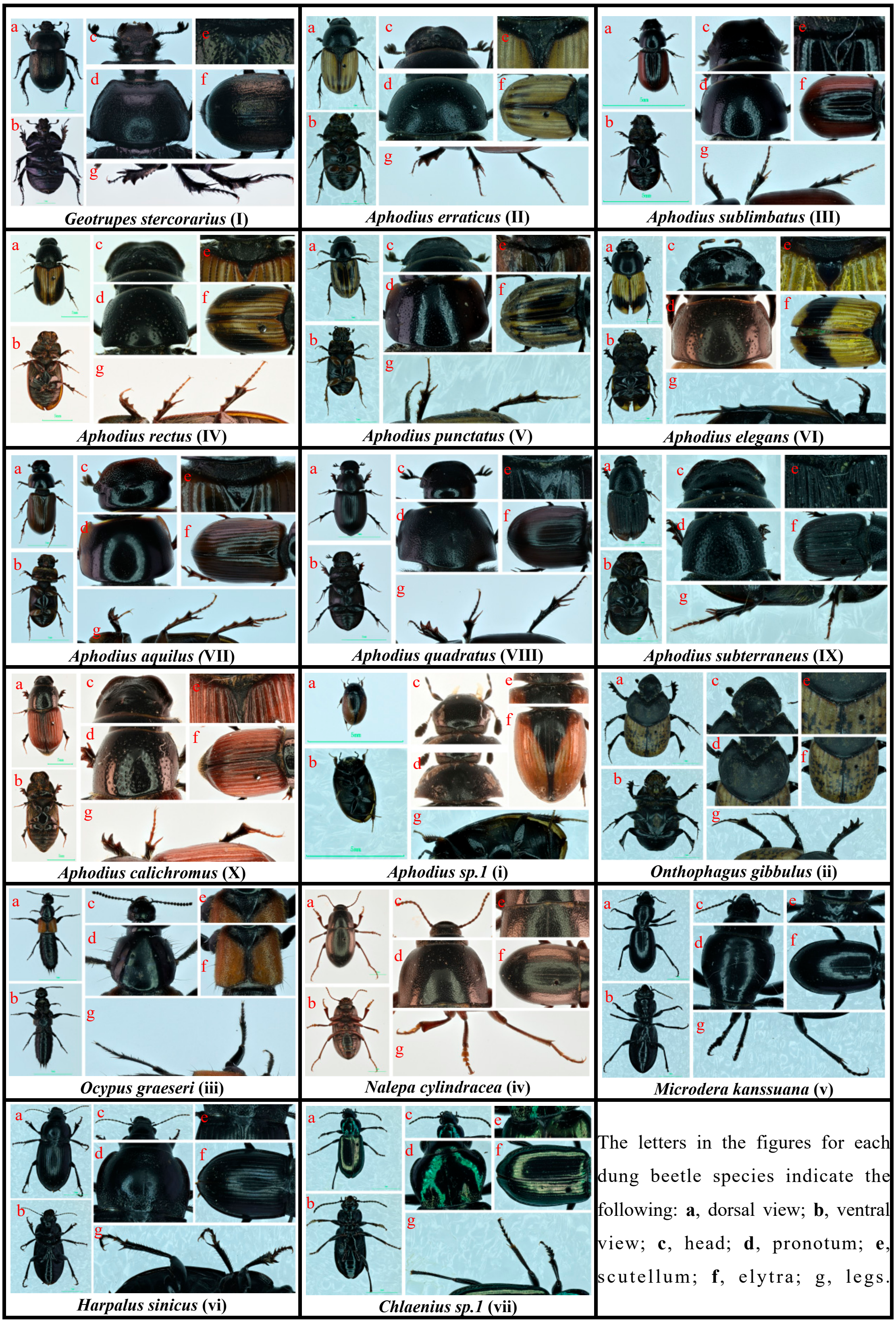

The letters in the figures for each dung beetle species indicate the following: **a**, dorsal view; **b**, ventral view; **c**, head; **d**, pronotum; **e**, scutellum; **f**, elytra; g, legs.

Figure S2. Diagnostic images of the complete dung-associated beetle voucher inventory.

Table S2. Ecological attributes, life-history information, and analytical inclusion status of the 17 beetle taxa recorded in yak dung.

Taxon	Ecological / functional group	Life-history information and notes	Diversity & niche analyses
Geotrupes stercorarius	Coprophagous; tunneler	Adults and larvae feed on cattle and horse dung, commonly excavating vertical burrows beneath fresh dung pats and transporting dung into the burrows; subterranean development is prolonged, and larvae and/or adults may overwinter in the soil[41].	**Included**
Aphodius erraticus	Coprophagous; dweller	Adult overwintering and reproductive diapause have been reported; warming experiments on the Tibetan Plateau indicate that oviposition and larval development are sensitive to temperature change[41].	**Included**
Aphodius sublimbatus	Coprophagous; dweller	Adult overwintering, summer reproduction, and the emergence of new adults have been reported; the species is widely distributed in East Asia and northern China[43].	**Included**
Aphodius rectus	Coprophagous; dweller	Adults and larvae can inhabit cattle dung; overwintered adults have been reported to become active in spring, reproduce in May–June, and produce a new adult cohort in autumn[66].	**Included**
Aphodius punctatus	Coprophagous; dweller	Identification references record the species from China, Korea, and Japan[43].	**Included**
Aphodius elegans	Coprophagous; dweller	Population studies have reported larval overwintering, adult aestivation, and autumn reproduction; the species has been recorded from Gansu, Qinghai, Tibet, and other regions of China[43].	**Included**
Aphodius aquilus	Coprophagous; dweller	Identification references record the species from Fujian, Hubei, Inner Mongolia, Sichuan, Shaanxi, Yunnan, Gansu, Qinghai, and other regions of China[43].	**Included**
Aphodius quadratus	Coprophagous; dweller	Identification references explicitly describe the species as coprophagous and record it from Liaoning, Shanxi, and other parts of Northeast Asia[67].	**Included**
Aphodius subterraneus	Coprophagous; dweller	Identification references record the species from Gansu, Qinghai, and other regions[67].	**Included**
Aphodius calichromus	Coprophagous; dweller	Identification references record the species from Guangxi, Fujian, Sichuan, and Yunnan[43].	**Included**
Aphodius sp. 1	Coprophagous; dweller	A small morphospecies (approximately 3–4 mm) collected from yak dung in the Qilian Mountains and observed feeding on yak dung.	**Included**
Onthophagus gibbulus	Coprophagous; tunneler	Feeds on animal dung; identification references report adult activity from May to August[41].	**Included**

Taxon	Ecological / functional group	Life-history information and notes	Diversity & niche analyses
Ocypus graeseri	Saprophagous rove beetle	Identification references record the species from Ningxia, Qinghai, Sichuan, and other regions; it is a non-scarabaeoid taxon and was retained only as a voucher record[42].	**Excluded**
Carabidae sp. 1	Predatory carabid	A non-scarabaeoid dung-associated taxon retained only as a voucher record.	**Excluded**
Harpalus sp. 1	Predatory carabid	A non-scarabaeoid dung-associated taxon retained only as a voucher record.	**Excluded**
Microdera kanssuana	Omnivorous tenebrionid	Identification references record the species from Wuwei, Gansu; it is a non-scarabaeoid taxon and was retained only as a voucher record.	**Excluded**
Nalepa cylindracea	Omnivorous tenebrionid	Identification references record the species mainly from high-elevation regions of Qinghai, Sichuan, and Tibet; it is a non-scarabaeoid taxon and was retained only as a voucher record.	**Excluded**

Note: "Included" indicates taxa used in both diversity analyses and niche calculations (12 taxa in total); "Excluded" indicates the five non-scarabaeoid dung-associated taxa (*Ocypus graeseri*, Carabidae sp. 1, *Harpalus* sp. 1, *Microdera kanssuana*, and *Nalepa cylindracea*) that were not used in these analyses.
